# High Diversity of Long Terminal Repeat Retrotransposons in Compact Vertebrate Genomes: Insights from Genomes of *Tetraodontiformes*

**DOI:** 10.3390/ani14101425

**Published:** 2024-05-10

**Authors:** Bingqing Wang, Ahmed A. Saleh, Naisu Yang, Emmanuel Asare, Hong Chen, Quan Wang, Cai Chen, Chengyi Song, Bo Gao

**Affiliations:** 1 College of Animal Science and Technology, Yangzhou University, Yangzhou 225009, China; mx120220902@stu.yzu.edu.cn (B.W.); elemlak1339@gmail.com (A.A.S.); dx120180101@yzu.edu.cn (N.Y.); asare.emmanuel175@yahoo.com (E.A.); mx120230878@stu.yzu.edu.cn (H.C.); mx120230886@stu.yzu.edu.cn (Q.W.); 007302@yzu.edu.cn (C.C.); cysong@yzu.edu.cn (C.S.); 2 Animal and Fish Production Department, Faculty of Agriculture (Al-Shatby), Alexandria University, Alexandria 11865, Egypt

**Keywords:** fish genomes, integrase, evolutionary dynamics, reverse transcriptase, superfamily

## Abstract

**Simple Summary:**

Long terminal repeat retrotransposons (LTR-RTNs) are vital in genome evolution and diversity. The compact genomes of *Tetraodontiformes* provide an excellent model for studying LTR-RTN dynamics. An analysis of the genomes of ten tetraodontiform species revealed a total of 819 full-length LTR retrotransposon sequences classified into nine families spanning four distinct superfamilies. Among them, the Gypsy superfamily displayed the highest level of diversity. *Takifugu* stood out for having the highest abundance of LTR families and sequences. Evidence of recent LTR-RTN activity and multiple invasions was observed in specific tetraodontiform genomes. This investigation provides valuable insights into the evolution of LTR retrotransposons and their impact on the structure and evolution of compact tetraodontiform genomes.

**Abstract:**

This study aimed to investigate the evolutionary profile (including diversity, activity, and abundance) of retrotransposons (RTNs) with long terminal repeats (LTRs) in ten species of *Tetraodontiformes*. These species, *Arothron firmamentum*, *Lagocephalus sceleratus*, *Pao palembangensis*, *Takifugu bimaculatus*, *Takifugu flavidus*, *Takifugu ocellatus*, *Takifugu rubripes*, *Tetraodon nigroviridis*, *Mola mola*, and *Thamnaconus septentrionalis*, are known for having the smallest genomes among vertebrates. Data mining revealed a high diversity and wide distribution of LTR retrotransposons (LTR-RTNs) in these compact vertebrate genomes, with varying abundances among species. A total of 819 full-length LTR-RTN sequences were identified across these genomes, categorized into nine families belonging to four different superfamilies: ERV (Orthoretrovirinae and Epsilon retrovirus), Copia, BEL-PAO, and Gypsy (Gmr, Mag, V-clade, CsRN1, and Barthez). The Gypsy superfamily exhibited the highest diversity. LTR family distribution varied among species, with *Takifugu bimaculatus*, *Takifugu flavidus*, *Takifugu ocellatus*, and *Takifugu rubripes* having the highest richness of LTR families and sequences. Additionally, evidence of recent invasions was observed in specific tetraodontiform genomes, suggesting potential transposition activity. This study provides insights into the evolution of LTR retrotransposons in *Tetraodontiformes*, enhancing our understanding of their impact on the structure and evolution of host genomes.

## 1. Introduction

Long terminal repeat (LTR) retrotransposons (LTR-RTNs) are a specific type of repetitive DNA sequence widely spread throughout the genomes of many organisms [1]. These retrotransposons are characterized by their distinctive structure, consisting of two identical regions at their ends known as LTRs [2]. LTR-RTNs consist of three regions: unique 3′ end (U3), repeated (R), and unique 5′ end (U5). LTRs consisting of (U3-R-U5) portions are important elements of retroviruses and related retrotransposons. LTRs encode the Polymerase protein (Pol) with essential domains, including reverse transcriptase (RT), ribonuclease H (RH), Protease (PR), and integrase (INT). The RT domain performs reverse transcription and is used for phylogenetic classification. LTR-RTNs also encode a related gag-like protein for nucleic acid binding and an envelope-like (env) fragment for potential retroviral transmission. They play a significant role in genome evolution and various biological processes [3,4,5]. LTR-RTNs play a significant role in the evolution and genetic diversity of genomes [6,7,8,9]. Additionally, LTR-RTNs contribute to the regulation of gene expression by acting as promoters or enhancers [10]. Understanding the biology and impact of LTR-RTNs can provide valuable insights into the dynamic nature of genomes and their evolution [3,11,12].

The compact genome size observed in tetraodontiform species indicates a significant genome reduction, which consequently entails the loss of non-essential genetic material [13,14,15,16]. The evolution of LTR-RTNs in compact genomes of vertebrates is a topic of great interest due to its unique characteristics [3,11,12]. Worth mentioning, the tetraodontiform order is known for having the smallest genome among vertebrates. For example, species of Pufferfish from the tetraodontiform order [17,18,19], such as tetraodon2 and fugu1, possess the most compact genomes among all vertebrates, with a size of approximately 350–400 Mb, which is roughly one-eighth of the size of the human genome [20,21]. The compact genomes of tetraodontiform species provide an excellent model for studying the evolutionary dynamics of LTR-RTNs [13,14,15,16]. On the other hand, the study of the evolution of LTR-RTNs in tetraodontiform species presents an opportunity to unravel the mechanisms behind compact genome evolution [22,23,24]. Furthermore, studies on the evolution of LTR-RTNs in tetraodontiform species can contribute to our understanding of the functional impact of repetitive elements in vertebrate genomes [20].

In this study, we aimed to identify and characterize the LTR-RTNs in the genomes of *Tetraodontiformes*. We systematically examined their diversity, distribution, abundance, structure, and evolutionary dynamics. Our investigation shed light on the evolution and evolutionary significance of LTR-RTNs in *Tetraodontiformes*, providing insights into their potential roles in host genome evolution and adaptation.

## 2. Material and Methods

### 2.1. Genomes Used and LTR Retrotransposon Mining

Ten genomes of *Tetraodontiformes* were retrieved from the NCBI database (https://www.ncbi.nlm.nih.gov, accessed on 25 June 2023). These genomes comprise eight compact genomes from the Tetraodontidae order (*Arothron firmamentum*, *Lagocephalus sceleratus*, *Pao palembangensis*, *Takifugu bimaculatus*, *Takifugu flavidus*, *Takifugu ocellatus*, *Takifugu rubripes*, and *Tetraodon nigroviridis*), along with two relatively large genomes from *Mola mola* and *Thamnaconus septentrionalis*, which belong to a closely related group to Tetraodontidae [25]. The Fish Tree of Life was developed utilizing the Common Taxonomy Tree (https://www.ncbi.nlm.nih.gov/Taxonomy/CommonTree/wwwcmt.cgi, accessed on 28 July 2023) and iTOL (https://itol.embl.de/itol.cgi, accessed on 29 July 2023) platforms, with species details sourced from NCBI’s Genome List database (https://www.ncbi.nlm.nih.gov/genome/browse, accessed on 28 July 2023).

LTR-RTNs were identified in the genomes of ten fish species using the LTRharvest v1.5.10 program [26]. Only LTR-RTNs with lengths ranging from 4 kb to 10 kb were kept. The left and right flanks (4 kb) of these LTR-RTNs were extended using the Bedtools slop program (https://bedtools.readthedocs.io/en/latest/content/tools/slop.html, accessed on 1 July 2023) to obtain the full-length sequences. Subsequently, the protein encoded by these LTR-RTNs was translated using the Bioedit software v7.2.0 (https://bioedit.software.informer.com/7.2, accessed on 16 August 2023). Only LTR-RTNs that encoded proteins longer than 500 amino acids were retained.

To identify the RT domains in the proteins encoded by LTR-RTNs, the RT domain sequences from the Pfam database (https://www.ncbi.nlm.nih.gov/pubmed/24288371, accessed on 22 August 2023) were used to constructed a hidden Markov model (HMM) profile (RT.hmm, Appendix S1), and then the hmmsearch tool in HMMER 3.4 (http://hmmer.org, accessed on 25 August 2023) was used to extract RT domains using RT.hmm. These LTR-RTNs encoding proteins more than 500 aa in length and harbouring RT domains were then clustered using the Vsearch program, with a 50% identity threshold [27]. For clusters consisting of three or more sequences, FastPCR v6.3 software was employed to identify the left- and right-end LTRs on both sides of the alignments in combination with the LTRharvest program [26]. Manual verification was performed to ensure accuracy. The obtained end LTRs were extracted and aligned to the remaining clusters (containing less than 3 sequences) to define their end LTRs by using the MAFFT program (https://mafft.cbrc.jp/alignment/software, accessed on 6 September 2023). Finally, the full-length LTR-RTNs that possess LTRs at both ends, encode proteins longer than 500 amino acids, and harbour RT domains were retained for further analysis.

### 2.2. Structure and Sequence Analysis of Retrotransposons and Proteins

Gag, Pol, and Env protein sequences were collected from the Pfam and NCBI databases for the construction of hmm models (Gag.hmm, Pol.hmm, and Env.hmm, Appendices S2–S4). These models were then used to extract the homologous protein sequences encoded by the identified LTR-RTNs. To detect the INT, RT, and RH domains, the online hmmscan program (https://www.ebi.ac.uk/Tools/hmmer/search/hmmscan, accessed on 11 October 2023) in conjunction with the NCBI Conserved Domains website (https://www.ncbi.nlm.nih.gov/Structure/cdd/wrpsb.cgi, accessed on 11 October 2023) was employed. Information such as the copy number of each LTR retrotransposon family and the length of various structures was documented.

To generate structural diagrams of LTR-RTNs, the IBS website (http://ibs.biocuckoo.org, accessed on 16 October 2023) was used. Protein or domain sequence similarity for each family was calculated using the Bioedit v7.2.0 software, and a heat map showing the similarities of Pol, RT, and catalytic “Asp-Asp-Glu” (DDE) proteins/domains was created using GraphPad v8.0.2 software. Additionally, the Jalview v2.11.3.2 software was utilized to generate a sequence alignment graph.

### 2.3. Construction of Phylogenetic Tree

To construct the phylogenetic tree, we obtained the reference sequences containing the reverse transcriptase (RT) domains from the NCBI database, which are widely recognized for their use in the phylogenetic analysis and classification of LTR-RTNs. The G-INS-I method of MAFFT was utilized to perform a multiple sequence alignment using the Pol proteins. Subsequently, the phylogenetic tree was constructed using the alignment of Pol proteins through the maximum likelihood method in the IQ-TREE program (http://www.iqtree.org, accessed on 25 October 2023). ModelFinder was employed to select the most suitable amino acid substitution model, and the ultrafast bootstrap approach with 1000 replicates was applied.

### 2.4. Evolution Activity of Retrotransposon

In *Tetraodontiformes*, the evolutionary dynamics of LTR-RTNs were assessed by estimating the insertion times of individual elements using the calcDivergenceFromAlign.pl tool within the RepeatMasker program [28]. This estimation utilized representative sequences for each element. The insertion time for each element was calculated according to the formula t = K/2r [29], where “t” signifies the insertion time in millions of years, “K” represents the divergence “k”, and “r” denotes the neutral mutation rates of transposable elements (TEs). An average substitution rate (r) of 1 × 10^−8^ substitutions per synonymous site per year was applied [30].

### 2.5. Theory/Calculation

The theory of the present study focuses on the identification and characterization of LTR-RTNs in the genomes of *Tetraodontiformes*. Our systematic analysis explores the diversity, distribution, abundance, structure, and evolutionary dynamics of these LTR-RTNs, providing insights into their potential roles in host genome evolution and adaptation. The Calculation section outlines our practical approach, which involved retrieving ten genomes of *Tetraodontiformes*, identifying LTR-RTNs using the LTRharvest program, extending their flanks, translating encoded proteins, and identifying reverse transcriptase domains. The resulting LTR-RTNs that met specific criteria were used for further analysis and exploration.

## 3. Results 

### 3.1. LTR-RTN Mining in Tetraodontiformes

**Figure 1:**
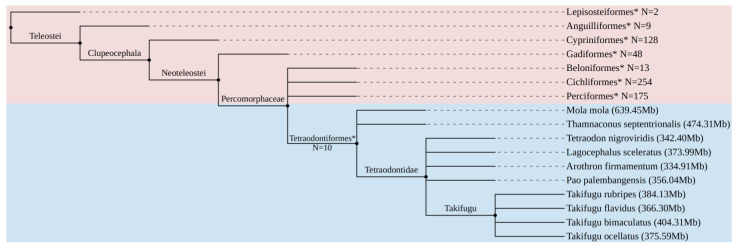
The Fish Tree of Life showcases 8 meticulously annotated representative fish genomes [34], with the blue section highlighting the condensed genomes of the *Tetraodontiformes*. The genomes of eight representative fish species are indicated by an asterisk, with N denoting the number of species within each genome. The number in parentheses denotes the genome size of the 10 tetraodontiform species.

LTR-RTNs were screened in the genomes of ten species from the tetraodontiform order, which is known for having relatively small genomes compared to other vertebrate species (Figure 1). Among these species, the Tetraodontidae family is particularly notable for its compacted genomes and well-annotated genes [31,32,33]. Using the LTRharvest v1.5.10 program, a total of 22,006 LTR-RTNs were identified in the tetraodontiform genomes, with 6163 of them ranging from 4000 to 10,000 base pairs in length. Following the filtering protocol detailed in the methods section, we ultimately obtained 819 full-length LTR-RTNs. These retrotransposons were distinguished by the presence of LTRs at both ends, encoded proteins exceeding 500 amino acids in length, and contained RT domains (Table 1).

### 3.2. Classification and Structure Organization of LTR-RTNs in Tetraodontiformes

**Figure 2:**
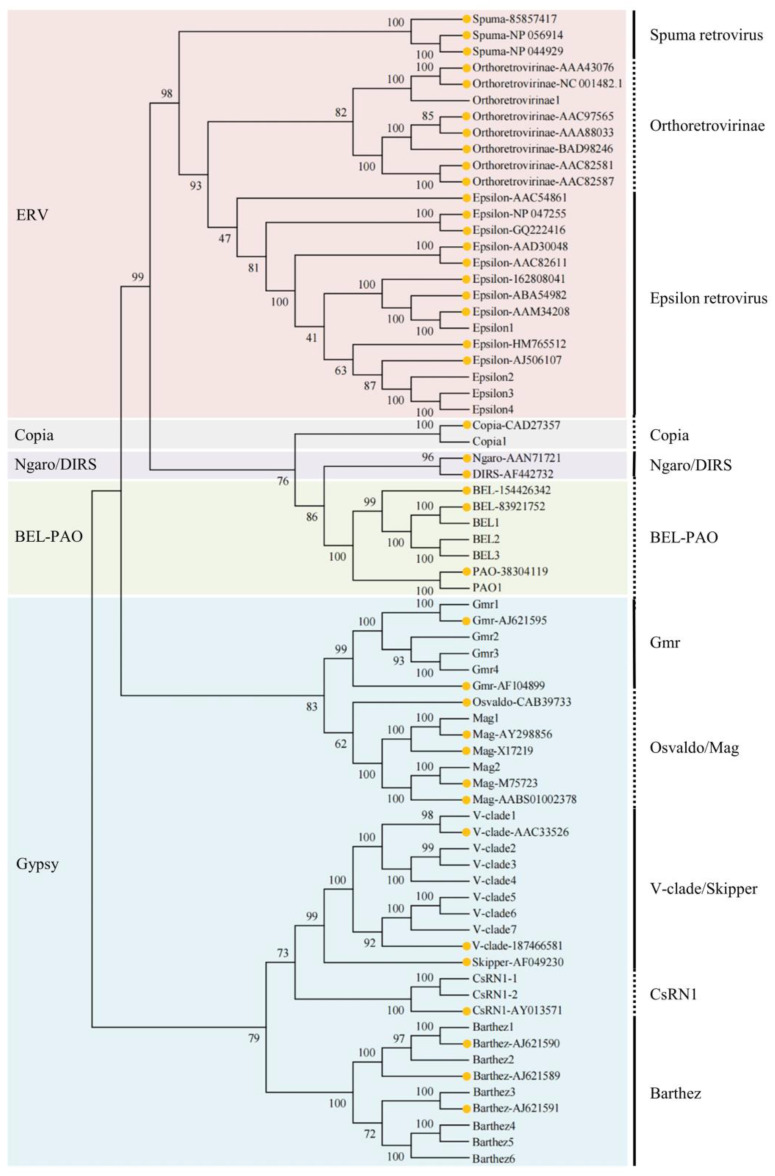
Phylogenetic analysis of LTR-RTNs in *Tetraodontiformes* based on the Pol protein sequences. Reference sequences with GenBank Accession Numbers were downloaded from NCBI and are highlighted with yellow dots.

Based on the phylogenetic tree (Figure 2), the structural characteristics, and LTR sequences (Table 2), the obtained 819 LTR sequences were classified into 31 LTR elements, belonging to nine families of four superfamilies: endogenous retrovirus “ERV” (Orthoretrovirinae and Epsilon retrovirus), Copia, BEL-PAO, and Gypsy (Gmr, Mag, V-clade, CsRN1, and Barthez). Gypsy represents the highest diversity, with five families of LTR-RTNs (Gmr, Mag, V-clade, CsRN1, and Barthez) detected, and the highest abundances, with 278 LTR sequences detected. V-clade and Epsilon retrovirus represent two of the most abundant families, each comprising over 100 LTR-RTN sequences. Within these families, V-clade1 and Epsilon2 had the highest number of LTR-RTN sequences, with 55 and 105, respectively. Some families, such as Copia and Orthoretrovirinae, had only one type of LTR retrotransposon, with a total of 3–4 retrotransposon sequences per element (Table 2).

**Figure 3:**
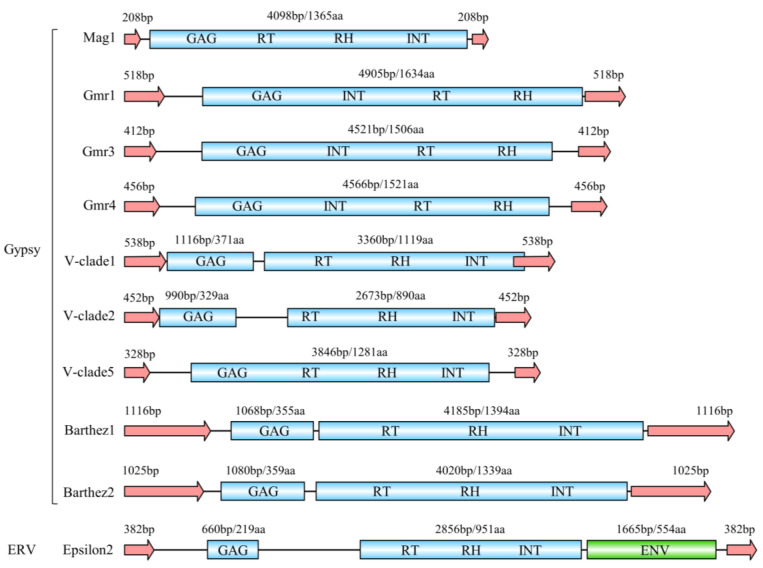
Structural characteristics and protein composition of 10 representative LTR-RTNs in studied tetraodontiform genomes. The red arrows represent LTRs. Gag: group-specific antigen protein; RT: reverse transcriptase domains; RH: ribonuclease domain; INT: integrase domain; ENV: envelope protein.

The lengths of the identified representative sequences of LTR-RTNs range from 4337 to 9854 bp. The Mag and CsRN1 retrotransposons are generally shorter, around 4000 bp. The LTRs have an approximate length of 200–1100 bp, with the majority falling within the range of 400–700 bp. The Mag and CsRN1 LTRs are shorter, approximately 200–300 bp, while certain Barthez families have LTRs longer than 1000 bp. Most LTR retrotransposon gag proteins have a length of 300–500 amino acids, but specific families, such as Barthez6 and Epsilon1, have gag proteins exceeding 700 amino acids. The length of pol proteins typically ranges from 800 to 1600 amino acids, while the env protein in the Epsilon retrovirus family is approximately 500–1300 amino acids long (Table 2 and Figure 3). Full-length LTR-RTNs consist of gag and pol proteins, while retroviruses of the Epsilon retrovirus family also include an Env protein. The presence of Gag, Pol, and Env proteins varies among different LTR retrotransposon families. Most families contain gag and pol proteins, but CsRN1, BEL-PAO, and Orthoretrovirinae families often lack gag proteins. Most LTR-RTNs have separate gag and pol proteins, while all Gmr and Mag retrotransposons have a continuous fusion of gag and pol proteins in a single ORF (Table 2 and Figure 3). More details about LTR-RTNs are presented in Table S1. The structural diagrams of ten representative LTR-RTNs were generated using the IBS website and are shown in Figure 3. The pol protein consists of an INT, RT and RH. In the V-clade1 element, the pol protein partially overlaps with the LTR, while proteins from other families are independent and do not overlap with the LTR (Figure 3).

### 3.3. Distribution of LTR Families in Compact Genomes of Vertebrates

Significant variations in the distribution of LTR families among different species were observed in the ten concentrated fish genomes. The highest abundance and diversity of LTR families were detected in the genomes of *Takifugu bimaculatus*, *Takifugu flavidus*, *Takifugu ocellatus*, and *Takifugu rubripes species*, all of which contained over 15 LTR elements. *Pao palembangensis* and *Thamnaconus septentrionalis species* had the second-highest number of detected LTR families, with three LTR elements each. The lowest abundance of LTR elements was found in the genomes of *Arothron firmamentum*, *Lagocephalus sceleratus*, *Mola mola*, and *Tetraodon nigroviridis* species, with only one LTR element detected in each genome. Different LTR retrotransposon families exhibit varying levels of dissemination in fish genomes. Among the four LTR superfamilies (Gypsy, ERV, BEL-PAO, and Copia), the Gypsy superfamily showed the widest distribution and the highest number of families among the 10 fish genomes, which is distributed in 9 fish genomes except *Mola mola*. The ERV superfamily was the next most widely distributed, present in 6 fish genomes. The BEL-PAO superfamily was found only in *Takifugu bimaculatus*, *Takifugu flavidus*, *Takifugu ocellatus*, and *Takifugu rubripes*, the four species with the highest abundance of the LTR elements. The Copia group did not show significant amplification in the 10 genomes of the fish, with only one element detected in *Takifugu flavidus*, *Takifugu rubripes*, and *Thamnaconus septentrionalis* (Table 3).

### 3.4. Protein Sequence Analysis

**Figure 4:**
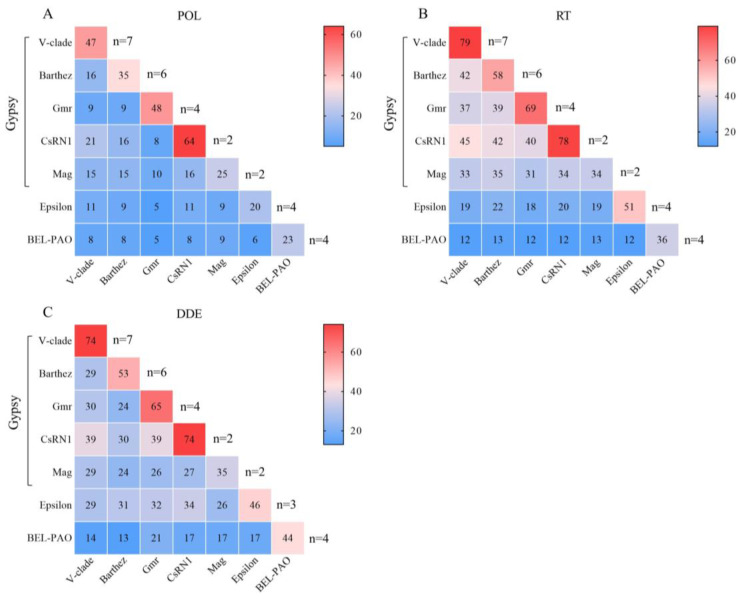
Sequence homology analysis and protein conservation patterns of LTR-RTNs in Tetraodontiform genomes. The sequence similarity of POL, RT, and DDE proteins is illustrated across various families of LTR retrotransposons in *Tetraodontiformes*, denoted as (**A**, **B**, and **C**), respectively. The heatmap values represent the average percentage similarity between protein families in the corresponding rows and columns, with “*n*” indicating the number of sequences for each family listed in the left column.

The sequence similarities of the POL (A), RT (B), and DDE (C) proteins among various families of LTR-RTNs in the fish condensed genome is depicted in a heatmap and summarized in Figure 4. The numbers in the heatmap represent the average percentage similarity of sequences between two categories in the corresponding rows and columns, with “n” denoting the number of sequences. Figure 4A illustrates the similarity of POL protein sequences in different families within the fish condensed genome. The Gypsy group displays relatively higher similarity compared to the BEL-PAO and ERV groups, with an average sequence similarity ranging from 25% to 64% within each family. Conversely, the BEL-PAO and ERV groups exhibit greater genetic diversity in their LTR-RTNs, with an average sequence homogeneity of 23% and 20%, respectively. These findings suggest that the BEL-PAO and ERV groups may represent older families. Additionally, the sequence homogeneity between different families is generally low. Contrasting the POL protein, the RT and DDE proteins demonstrate a higher level of conservation. Figure 4B,C illustrate the RT and DDE structures of the V-clade, Gmr, and CsRN1 within the Gypsy group, highlighting their elevated sequence homogeneity. Conversely, the Barthez, Mag, Epsilon, and BEL-PAO families exhibit greater divergences in their RT and DDE sequences.

**Figure 5:**
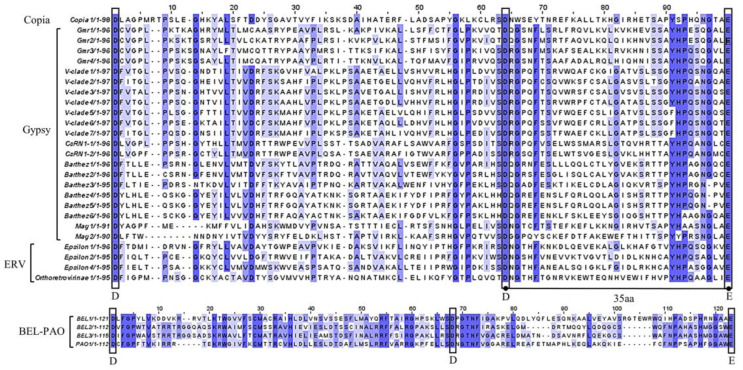
The comparative analysis of the DDE domain in Pol proteins. The black box illustrates the conservative amino acid “DDE” structure. In the Copia, Gypsy, and ERV superfamilies, the distance between the second “D” and the third “E” is 35 amino acids.

Figure 5 shows the comparative analysis of the DDE domain of integrase, where the black boxes represent conserved amino acids forming the “DDE” motif. Integrases of LTR-RTNs typically possess a catalytic domain composed of a triad motif consisting of D (aspartic acid), D, and E (glutamic acid). This motif interacts with divalent cations (Mg^+2^ or Mn^+2^) and catalyses the cleavage of DNA on both sides of the retrotransposon, facilitating its movement to a new location, which is crucial for retrotransposition [35]. The DDE motif is typically highly conserved, especially the amino acid distance between the second “D” and the third “E” [35,36], as the enzymatic activity of the transposase relies on their presence and relative positioning within the active site [37,38]. As depicted in Figure 5, the DDE structure is highly conserved across different families. The distance between the second “D” and the third “E” is 35 amino acid residues in the Copia, Gypsy, and ERV groups, indicating a potential functional domain. However, the BEL-PAO group shows higher heterogeneity in the amino acid distance between the second “D” and the third “E”, suggesting that they may be truncated or dysfunctional.

### 3.5. Evolution Dynamics of LTR in Compact Genomes of Vertebrates

**Figure 6:**
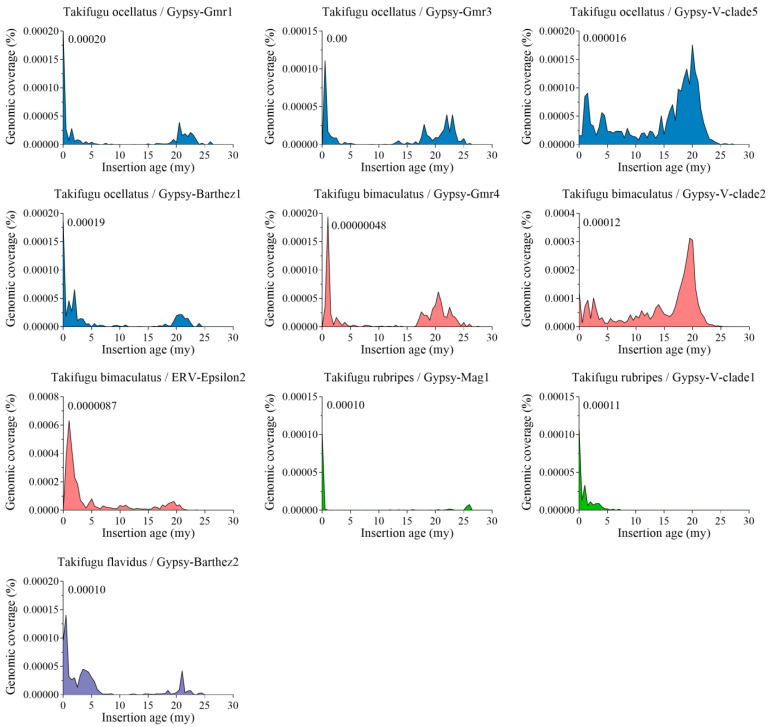
Evolutionary dynamics of LTR-RTNs in *Tetraodontiformes*: insights from insertion ages of 10 enriched LTR-RTNs. The *X*-axis represents the insertion age (millions of years; My), and the *Y*-axis represents the coverage (%) of each LTR-RTN in the genome. The number on the right side of the *Y*-axis indicates the genomic percentage with LTR-RTNs inserted at age 0.

The evolutionary dynamics of LTR-RTNs in fish genomes were investigated by analysing the insertion age. A total of 10 LTR-RTNs (Mag1, Gmr1, Gmr3, Gmr4, V-clade1, V-clade2, V-clade5, Barthez1, Barthez2, and Epsilon2) out of the 31 LTR-RTNs identified in *Tetraodontiformes* were chosen for evolutionary activity prediction in Figure 6, each containing 10 or more copies with over 60% being full-length LTR-RTNs encoding retrovirus proteins (gag and pol proteins). The analysis of insertion age of the remaining 21 LTR-RTNs is presented in Figure S1. The insertion age of the LTR-RTNs elements revealed differential evolutionary dynamics in vertebrates. Most LTR-RTN families exhibit relatively young insertion ages with recent and peak activities less than 5 million years ago, such as Barthez, Gmr, Mag, and BEL-PAO, indicating recent invasions in these species (Figure 6 and Figure S1). However, in the families of Epsilon retrovirus and V-clade, most LTR-RTNs were ancient insertions and underwent multiple waves of amplification. In Figure 6, Mag1, Gmr1, V-clade1, and Barthez1 displayed high activity peaks at insertion age 0, indicating they were very young invaders. Active retrotransposons tend to have relatively intact copies, and combined with the sequence identity analysis of Figure 4, Gmr1, V-clade1, and Barthez1 may possess transpositional activity.

## 4. Discussion

### 4.1. Diversity

Tetraodontiform species are an order of vertebrates with the smallest genomes, making them valuable for studying LTR elements in the context of genome size evolution [20]. In the present study, the genome size for the investigated species ranged from 334.905 to 639.452 Mb for *Arothron firmamentum* and *Mola mola* species, respectively (Table 1). This agrees with [21,39], who reported that Pufferfish have genome sizes of less than 400 Mb, while salmon have genome sizes exceeding 3000 Mb. The underlying reasons behind this significant variation in genome size remain largely undisclosed. Additionally, a study by [40] confirmed a notable range in genome sizes within Ray-Finned Fishes, spanning from 1194.360 to 9111.360 Mb, primarily influenced by LTRs and other transposable elements (TEs) that play a crucial role in shaping species diversity.

On the other side, analysing the LTR content in the genomes of these fishes provides insights into the role of these elements in shaping the genetic architecture of vertebrates [8,21]. Comparative studies also contribute to our understanding of LTR evolution patterns in compact vertebrate genomes [9]. In the present study, we examined the diversity, activity, and abundance of LTR-RTNs in the tetraodontiform group. Previous research has identified six groups of LTRs (BEL/PAO, Copia, DIRS, Ngaro, Gypsy, and ERV) in teleost genomes [21]. We found that four groups of LTRs (ERV, Copia, BEL-PAO, and Gypsy) were present in these specific fish genomes, while DIRS and Ngaro were not detected. Regarding the LTR-RTNs obtained from FishTEDB (https://www.fishtedb.com/project/species, accessed on 18 April 2024), the LTR-RTNs of the Nargo/DIRs family were all short or decayed and were filtered based on a stringent standard protocol. As described in Section 2.1, only LTR-RTNs with lengths between 4 kb to 10 kb were retained for further analysis. It is worth noting that our exclusion criteria may have restricted the identification of LTR-RTNs in genomes, as the study specifically focused on potential functional LTR-RTNs with long LTRs and protein-encoding capacity. Therefore, further investigation of these genomes with short LTRs may be required.

In the previous study, the Gypsy superfamily was shown to be highly diverse, consisting of five branches (Gmr, Mag, V-calde, CsRn1, and Barthez), which were found in teleost species [21]. In the present study of tetraodontiform species, the identification of five families and 21 LTR-RTNs of the Gypsy superfamily aligns with previous research, highlighting its substantial impact on vertebrate genomes. The presence of multiple branches within Gypsy suggests its dynamic evolution and potential contribution to genome complexity [2,13]. ERV was distributed in six species, which also implies its significant impact on genome evolution in *Tetraodontiformes*. On the other hand, the smaller superfamily structures observed in BEL-PAO and Copia indicate their relatively limited impact on tetraodontiforme genomes compared to Gypsy and ERV.

### 4.2. Distribution and Abundances

Comparative analyses of LTR element distribution aid in deciphering underlying factors influencing their evolutionary dynamics, and investigating the effect of LTR element domestication uncovers novel genetic elements and regulatory mechanisms [40,41,42]. In the present study, we investigated the abundance and variety of LTR-RTN families in ten concentrated tetraodontiform genomes, revealing intriguing patterns and insights into LTR distribution dynamics. The analysis revealed substantial variations in the distribution of LTR families among the ten fish genomes. Notably, *Takifugu bimaculatus*, *Takifugu flavidus*, *Takifugu ocellatus*, and *Takifugu rubripes* species exhibited the highest richness in terms of the number and variety of LTR elements. Each of these four genomes contained over 15 distinct LTR elements, indicating a significant degree of genome expansion and diversification. *Pao palembangensis* and *Thamnaconus septentrionalis* species followed with a lower abundance of LTR elements, with three LTR-RTNs detected in each.

The copy number (abundance) varied significantly among the LTR-RTNs and families; only less than five copies were detected for some LTR-RTNs, such as Mag2, V-clade3, V-clade6, and V-clade7, while Epsilon2 had more than 100 copies. At the family level, V-clade and Epsilon retrovirus were the predominant types, with over 100 LTR-RTNs in each. V-clade1 and Epsilon2 had the highest numbers of LTR-RTNs, with 55 and 105, respectively. Some families displayed a low diversity and copy number, such as Copia and Orthoretrovirinae, which had only one type of LTR-RTN, and the copy number was three to four. These unique features of LTR-RTN evolution in the compact genomes of the studied species shed light on the evolution of small-genome vertebrate lineages [42,43].

### 4.3. Structure and Evolution Activity

Understanding the structural characteristics of LTR-RTNs in fish genomes contributes to elucidating their functional implications and evolutionary significance [9,13,15,24,44]. Several studies have reported that the structural organization of retrotransposons in compact vertebrate genomes involves the presence of LTRs flanking the retrotransposon DNA sequence. In most cases, the length of these LTRs can vary from approximately 200 to 1100 bp, with shorter LTRs observed in specific retrotransposon families such as Mag and CsRN1 ranging from 200 to 300 bp, consistent with our study [5,6,45]. It is revealed that LTR-RTNs have different structural characteristics, with most families containing gag and pol proteins but some lacking gag proteins, whereas retrotransposons of the Epsilon retrovirus type also include an env protein. Most LTR-RTNs encode separate gag and pol proteins, but interestingly, the Gmr and Mag families exhibited a continuous fusion of gag and pol proteins. The lengths of the LTR retrotransposon sequences range from 4337 to 9854 bp, with Mag and CsRN1 retrotransposons generally being shorter. The identification and characterization of these retrotransposons provide a foundation for further studies exploring their functional implications and evolutionary significance. Additionally, the variation in length and protein composition among different families suggests the existence of diverse mechanisms and evolutionary dynamics underlying LTR retrotransposon activity in fish genomes [9,13,15,24,44].

The similarity of Pol, RT, and DDE protein sequences varied among LTR retrotransposon families, with Gypsy showing higher sequence similarities compared to BEL-PAO and ERV families (Figure 4 and Figure 5). High numbers of full-length copies of LTR-RTNs were identified for some families of Gypsy, suggesting that they display recent and current activity. In brief, the variations in protein organization, LTR length, and protein–LTR interactions provide insights into the diversity and adaptability of these retrotransposon families [6,41,46,47].

Furthermore, the insertion age was analysed to assess the evolution activity of the identified LTR-RTNs, providing valuable insights into the evolutionary dynamics of retrotransposons and their host genomes. Some LTR-RTNs of the Gypsy superfamily exhibited a high sequence identity of the transposase enzyme (Figure 4 and Figure 5) and recent insertions (Figure 6 and Figure S1), both of which support their potential functional activity in certain species within *Tetraodontiformes*. However, almost all LTR-RTNs of the Epsilon retrovirus and V-clade families (except for V-clade1) exhibit multiple peaks of activity, with the majority of copies being ancient insertions. This may suggest repeated invasions of LTR-RTNs within the genome or a new life cycle due to horizontal transfer [48], which implies a complex evolutionary history involving multiple insertions and subsequent expansion events of LTR-RTNs.

## 5. Conclusions

In this study, 819 LTR-RTN sequences were identified in the compact tetraodontiform genomes, which were classified into nine families and four superfamilies, revealing a high diversity of LTR-RTNs within this order. The representative sequences of the LTR-RTNs had lengths ranging from 4337 to 9854 bp, with the Mag and CsRN1 retrotransposons generally being shorter. Variations in Pol, RT, and DDE protein sequence similarities were observed among the LTR retrotransposon families, with Gypsy displaying higher sequence similarities compared to the BEL-PAO and ERV superfamilies. The distribution of the LTR families differed among the fish species, with *Takifugu bimaculatus*, *Takifugu flavidus*, *Takifugu ocellatus*, and *Takifugu rubripes* exhibiting the highest richness. An evolutionary dynamics analysis indicated recent activity of some LTR-RTNs in certain species. The findings of this study provide insights into the evolutionary profile of LTR-RTNs in compact tetraodontiform genomes and contribute to our understanding of their impact on the evolution of small fish genomes.

## Figures and Tables

**Table 1:** LTR retrotransposons (LTR-RTNs) identified by LTRharvest in compact genomes of vertebrates.

Species	Common Name	LTR-RTNs Identified	LTR-RTNs <4 kb and <10 kb	Full-Length LTR-RTNs *	Ref. Genome	Genome Size
*Arothron firmamentum*	Starry Pufferfish	1224	405	3	GCA_016586285.1	334.905
*Lagocephalus sceleratus*	Silver-Cheeked Toadfish	3389	35	3	GCA_911728415.1	373.990
*Mola mola*	Ocean Sunfish	1125	381	63	GCA_001698575.1	639.452
*Pao palembangensis*	South Sumatran Puffer	1199	365	65	GCA_015343265.1	356.042
*Takifugu bimaculatus*	Two-Spot Pufferfish	3420	1062	192	GCA_004026145.2	404.312
*Takifugu flavidus*	Yellow Pufferfish	2783	937	124	GCF_003711565.1	366.303
*Takifugu ocellatus*	Ocellated Pufferfish	2367	678	117	GCA_027382335.1	375.589
*Takifugu rubripes*	Tiger Puffer	2757	861	186	GCF_901000725.2	384.127
*Tetraodon nigroviridis*	Green Spotted Pufferfish	1192	460	24	GCA_000180735.1	342.403
*Thamnaconus septentrionalis*	Northern Round Herring	2550	979	42	GCA_009823395.1	474.310
Total		22,006	6163	819		

* Note: full-length LTR-RTNs refer to the retrotransposons containing LTRs at both ends, encoding proteins exceeding 500 amino acids in length, and containing RT domains.

**Table 2:** Classification and characterization of LTR-RTNs in compact genomes of vertebrates.

Family	Element	Number of Sequences					Length of Sequence					
		Copy	Full LTR	Gag	Pol	Env	Consensus (bp)	LTR (bp)	Gag (aa)	Pol (aa)	Gag and Pol (aa)	Env (aa)
Gmr		47	41	40	47	0						
	Gmr1	20	16	19	20	0	6465	518	-	-	1634	-
	Gmr2	6	6	4	6	0	6058	337	-	-	1564	-
	Gmr3	11	10	9	11	0	6273	412	-	-	1506	-
	Gmr4	10	9	8	10	0	6223	456	-	-	1521	-
Mag		24	22	24	23	0						
	Mag1	20	18	20	19	0	4696	208	-	-	1365	-
	Mag2	4	4	4	4	0	4813	211	-	-	1330	-
V-clade		112	94	90	111	0						
	V-clade1	55	47	47	55	0	5558	538	371	1119	-	-
	V-clade2	29	25	25	28	0	5226	443	329	890	-	-
	V-clade3	3	3	2	3	0	5346	497	326	822	-	-
	V-clade4	5	3	1	5	0	6444	454	309	1016	-	-
	V-clade5	12	9	11	12	0	5369	328	-	-	1281	-
	V-clade6	4	3	1	4	0	5430	367	306	801	-	-
	V-clade7	4	4	3	4	0	5264	396	414	1119	-	-
CsRN1		39	38	0	39	0						
	CsRN1-1	21	21	0	21	0	4337	174	-	1061	-	-
	CsRN1-2	18	17	0	18	0	4552	302	-	1069	-	-
Barthez		56	38	30	56	0						
	Barthez1	13	11	11	13	0	7871	1116	355	1394	-	-
	Barthez2	12	9	8	12	0	7566	1025	359	1339	-	-
	Barthez3	4	2	0	4	0	8756	1114	-	1563	-	-
	Barthez4	5	5	0	5	0	7513	442	-	1580	-	-
	Barthez5	17	7	9	17	0	7544	259	526	1580	-	-
	Barthez6	5	4	2	5	0	7310	329	879	1137	-	-
BEL-PAO		24	20	0	22	0						
	BEL1	3	3	0	3	0	7624	728	-	1434	-	-
	BEL2	7	5	0	7	0	6587	536	-	1808	-	-
	BEL3	10	9	0	9	0	7341	652	-	1970	-	-
	PAO1	4	3	0	3	0	5939	456	-	1617	-	-
Copia	Copia1	4	4	2	4	0	4794	233	454	653	-	-
Orthoretrovirinae	Orthoretrovirinae1	3	3	0	3	0	8626	433	-	1108	-	-
Epsilon retrovirus		134	116	82	125	51						
	Epsilon1	21	19	11	14	9	8630	665	728	776	-	487
	Epsilon2	105	93	65	103	40	8158	382	219	951	-	554
	Epsilon3	3	2	2	3	0	7880	470	576	740	-	-
	Epsilon4	5	2	4	5	2	9854	377	604	985	-	477

**Table 3:** The distribution of LTR families among 10 studied species of *Tetraodontiformes*.

Superfamilies/Families	*Arothron firmamentum*	*Lagocephalus sceleratus*	*Mola mola*	*Pao palembangensis*	*Takifugu bimaculatus*	*Takifugu flavidus*	*Takifugu ocellatus*	*Takifugu rubripes*	*Tetraodon nigroviridis*	*Thamnaconus septentrionalis*
*Gypsy	1	1		3	15	14	12	16	1	1
Gmr		1			4	3	4	3		
Mag					1	1	1	2		
V-clade	1			3	4	4	3	4		
CsRN1					2	2	1	2	1	
Barthez					4	4	3	5		1
*ERV			1		3	2	3	2		1
Epsilon retrovirus					3	2	3	2		1
Orthoretrovirinae			1							
*BEL-PAO					3	2	3	4		
*Copia						1		1		1
Total	1	1	1	3	21	19	18	23	1	3

The symbol “*” represents superfamilies.

## Data Availability

All data generated or analysed during this study are included in the article and Appendices S1–S4 and Supplementary Files.

## Supplementary Materials

The following supporting information can be downloaded at https://www.mdpi.com/article/10.3390/ani14101425/s1, Table S1: Details of LTR retrotransposons (LTR-RTNs) found in Tetraodontiformes genome, including LTR-RTN sizes, LTR sizes and pair identity, motif sequences, sequence locations, and species; Figure S1: Evolutionary dynamics of LTR-RTNs in Tetraodontiformes: insights from insertion ages of LTR-RTNs. The *X*-axis represents the insertion age (My, millions of years), and the *Y*-axis represents the coverage (%) of each LTR-RTN in the genome. The number on the right side of the *Y*-axis indicates the genomic percentage with LTR-RTNs inserted at age 0.
