# Supplementary material for: High Diversity of Long Terminal Repeat Retrotransposons in Compact Vertebrate Genomes: Insights from Genomes of Tetraodontiformes
Source: Animals (Basel). 2024 May 10;14(10):1425. doi: 10.3390/ani14101425 (PMC11117352; doi:10.3390/ani14101425)
Supplement: Supplementary file 1 [file animals-14-01425-s001.zip › Supplementary Files/Supplementary File 2_Figure S1.pdf]

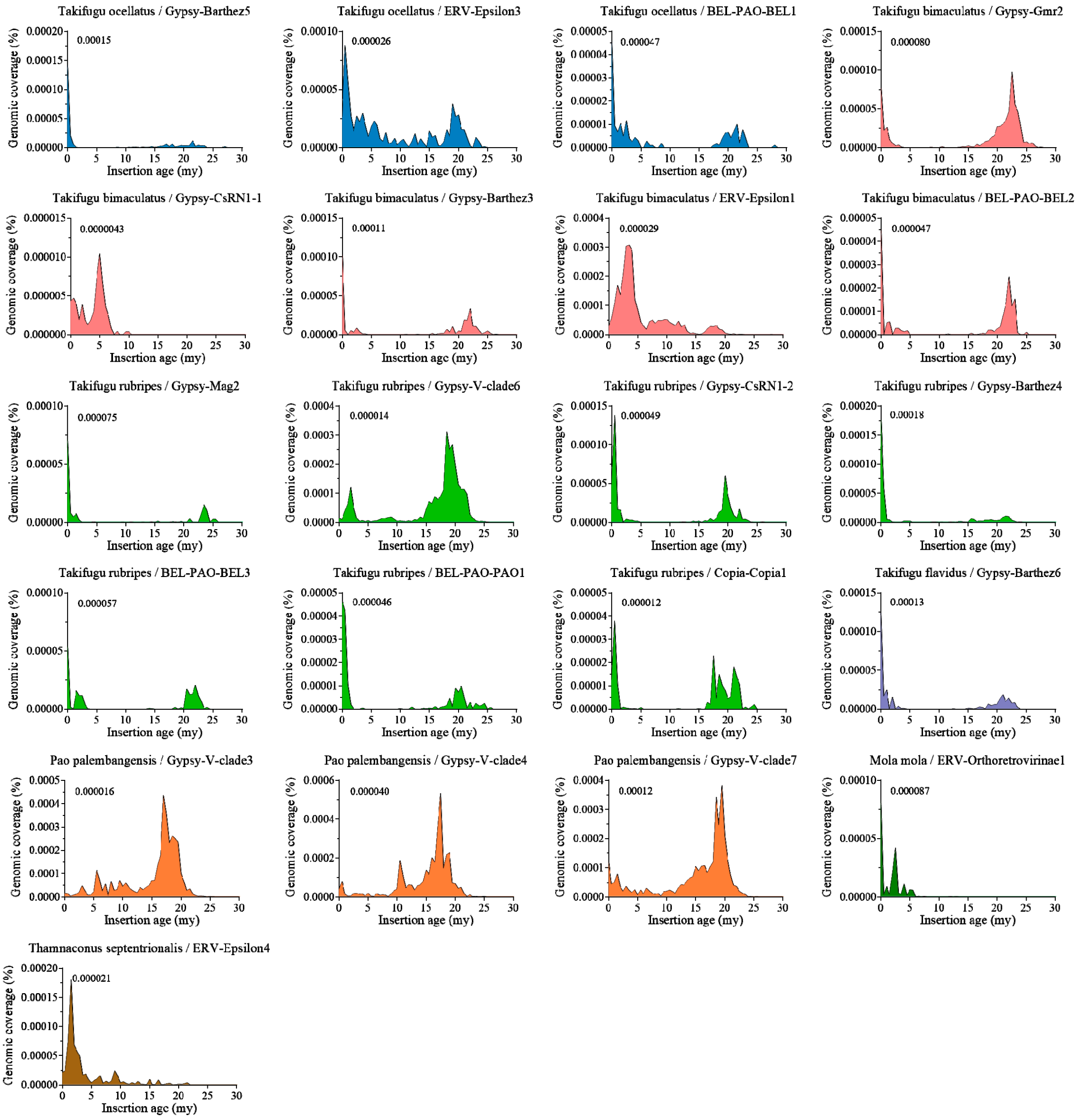

Figure S1. Evolutionary dynamics of LTR-RTNs in *Tetraodontiformes*: Insights from insertion ages of LTR-RTNs. The x-axis represents the insertion age (My, millions of years), and the y-axis represents the coverage (%) of each LTR-RTN in the genome. The number on the right side of the Y-axis indicates the genomic percentage with LTR-RTNs inserted at age 0.
